# Mitochondrial DNA copy number and incident atrial fibrillation

**DOI:** 10.1186/s12916-020-01715-6

**Published:** 2020-09-16

**Authors:** Di Zhao, Traci M. Bartz, Nona Sotoodehnia, Wendy S. Post, Susan R. Heckbert, Alvaro Alonso, Ryan J. Longchamps, Christina A. Castellani, Yun Soo Hong, Jerome I. Rotter, Henry J. Lin, Brian O’Rourke, Nathan Pankratz, John A. Lane, Stephanie Y. Yang, Eliseo Guallar, Dan E. Arking

**Affiliations:** 1 Departments of Epidemiology and Medicine, and Welch Center for Prevention, Epidemiology, and Clinical Research, Johns Hopkins University Bloomberg School of Public Health, 2024 E. Monument Street, Room 2-645, Baltimore, MD 21205 USA; 2 Departments of Biostatistics and Medicine, University of Washington, Seattle, WA USA; 3 Ciccarone Center for the Prevention of Heart Disease, Johns Hopkins University School of Medicine, Baltimore, MD USA; 4 Department of Epidemiology, University of Washington School of Public Health, Seattle, WA USA; 5 Department of Epidemiology, Rollins School of Public Health, Emory University, Atlanta, GA USA; 6 McKusick-Nathans Institute of Genetic Medicine, Johns Hopkins University School of Medicine, 733 N. Broadway, Miller Research Building, Room 459, Baltimore, MD 21205 USA; 7 Institute for Translational Genomics and Population Sciences and Department of Pediatrics, Los Angeles Biomedical Research Institute at Harbor-UCLA Medical Center, Torrance, CA USA; 8 Department of Medicine, Johns Hopkins University School of Medicine, Baltimore, MD USA; 9 Department of Laboratory Medicine and Pathology, University of Minnesota, Minneapolis, USA

**Keywords:** Mitochondria DNA copy number, mtDNA, Atrial fibrillation

## Abstract

**Background:**

Mechanistic studies suggest that mitochondria DNA (mtDNA) dysfunction may be associated with increased risk of atrial fibrillation (AF). The association between mtDNA copy number (mtDNA-CN) and incident AF in the general population, however, remains unknown.

**Methods:**

We conducted prospective analyses of 19,709 participants from the Atherosclerosis Risk in Communities Study (ARIC), the Multi-Ethnic Study of Atherosclerosis (MESA), and the Cardiovascular Health Study (CHS). mtDNA-CN from the peripheral blood was calculated from probe intensities on the Affymetrix Genome-Wide Human single nucleotide polymorphisms (SNP) Array 6.0 in ARIC and MESA and from multiplexed real-time quantitative polymerase chain reaction (qPCR) in CHS. Incident AF cases were identified through electrocardiograms, review of hospital discharge codes, Medicare claims, and death certificates.

**Results:**

The median follow-up time was 21.4 years in ARIC, 12.9 years in MESA, and 11.0 years in CHS, during which 4021 participants developed incident atrial fibrillation (1761 in ARIC, 790 in MESA, and 1470 in CHS). In fully adjusted models, participants with the lowest quintile of mitochondria DNA copy number had an overall 13% increased risk (95% CI 1 to 27%) of incident atrial fibrillation compared to those with the highest quintile. Dose-response spline analysis also showed an inverse association between mitochondria DNA copy number and hazard for atrial fibrillation for all three cohorts. These associations were consistent across subgroups.

**Conclusions:**

Mitochondria DNA copy number was inversely associated with the risk of AF independent of traditional cardiovascular risk factors. These findings implicate mitochondria DNA copy number as a novel risk factor for atrial fibrillation. Further research is warranted to understand the underlying mechanisms and to evaluate the role of mitochondria DNA copy number in the management of atrial fibrillation risk.

## Background

Atrial fibrillation (AF) is the most common form of clinical cardiac arrhythmia, with rising prevalence and incidence worldwide. The lifetime risk of developing AF ranges from 20 to 37% in Whites and Blacks [1–3], and it is estimated that the number of adults with AF will double in the USA by the year 2050, affecting more than 8 million people [4]. AF imposes considerable mortality and morbidity risks related to cardiovascular events and thromboembolism and is associated with tremendous healthcare costs [5, 6]. The high lifetime risk and adverse consequences of AF highlight the critical need for identifying novel risk markers that may provide insights into AF prevention and treatment.

Mitochondria generate energy for the cell through converting nutrients and oxygen into adenosine triphosphate (ATP) [7]. Unlike other organelles, mitochondria have their own circular DNA (mtDNA), which encodes essential genes for oxidative phosphorylation. Each cell contains on average 10^3^ to 10^4^ copies of mtDNA, with variations by cell type and development phase [8]. Mitochondrial DNA copy number (mtDNA-CN) is proportional to the transcription of mitochondrial genes and is a marker of mitochondrial dysfunction [9]. Indeed, reduced mtDNA-CN from the peripheral blood is associated with adverse cardiovascular disease (CVD) events including heart failure, all-cause mortality, sudden cardiac death, and atherosclerotic CVD [10–13], as well as with CVD risk factors, including hypertension, diabetes, atherosclerosis, and chronic kidney disease [14–16].

Emerging evidence from mechanistic studies suggests that mtDNA dysfunction may be associated with an increased risk of AF through reduced ATP production and elevated reactive oxygen species [17, 18]. The association between mtDNA-CN and incident AF in the general population, however, remains unknown. In the present study, we examined the prospective association between baseline mtDNA-CN and the risk of incident AF among participants from 3 community-based prospective cohort studies: the Atherosclerosis Risk in Communities (ARIC) study, the Multi-Ethnic Study of Atherosclerosis (MESA), and the Cardiovascular Health Study (CHS).

## Methods

### Study population

**Fig. 1:**
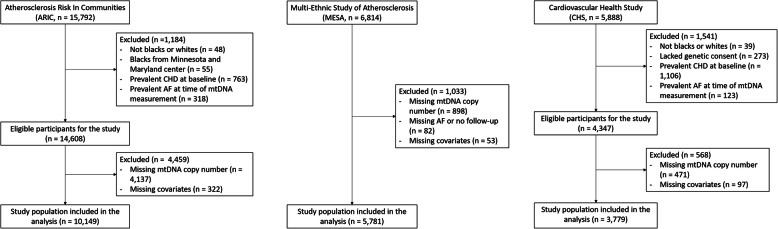
Flowchart of study participants

ARIC is a prospective cohort study of 15,792 men and women 45–64 years of age at baseline (1987–1989) [19]. Participants were randomly selected from 4 communities in the USA: Forsyth County, NC; Jackson, MS; Minneapolis suburbs, MN; and Washington County, MD. We excluded participants who reported race other than Black or White (*n* = 48), Blacks from the Minnesota and Maryland centers because the numbers are too small for adequate adjustment or within-community comparisons (*n* = 55), and participants with prevalent CHD (*n* = 667) or prevalent AF at the time of mtDNA-CN measurement (*n* = 292). We further excluded participants whose mtDNA-CN was not measured due to sample or measurement availability (*n* = 4259) and those missing other covariates (*n* = 322). The final sample included 10,149 participants (Fig. 1).

MESA is a prospective cohort study of 6814 men and women aged 45–84 years of age who were free of clinical cardiovascular disease (CVD) at the baseline visit (2000–2002) [20]. We excluded participants whose mtDNA-CN was not measured due to sample or technique availability (*n* = 898), who had no information on AF during follow-up (*n* = 82), or who were missing other covariates (*n* = 53). The final study sample included 5781 participants.

CHS is a prospective cohort study of 5888 men and women aged 65 years and older [21]. The original cohort of 5201 participants was recruited in 1989–1990 from random samples of Medicare eligibility lists, and an additional predominantly African-American cohort of 687 participants was recruited in 1992–1993. We excluded participants who reported race other than Black or White (*n* = 39), who lacked genetic consent (*n* = 273), and who had prevalent CHD (*n* = 1106) or prevalent AF at the time of mtDNA-CN measurement (*n* = 123). After further excluding participants whose mtDNA-CN was not measured due to sample or technique availability (*n* = 471), and those missing other covariates (*n* = 97), the final study sample included 3779 participants.

### mtDNA copy number

In ARIC, DNA for mtDNA-CN analysis was collected at visit 1 (1987–1989) for 389 participants (3.8%), at visit 2 (1990–1992) for 8221 participants (81.0%), at visit 3 (1993–1995) for 1485 participants (14.6%), and at visit 4 for (1996–1998) for 54 participants (0.5%). In MESA and CHS, DNA for mtDNA-CN analysis was collected at visit 1. The visit of DNA collection was considered as the baseline visit for each participant in the present analysis.

In ARIC and MESA, DNA samples were isolated from buffy coat and genotyped using Affymetrix Genome-Wide Human SNP Arrays 6.0 (the Genvisis software package [www.genvisis.org]) [11, 12, 22]. Mitochondrial SNPs were collected across all samples and were signaled with high-quality mitochondrial probes. Unadjusted mtDNA-CN was determined as the median of normalized probe intensity differences across all mitochondrial SNPs. To correct for technical artifacts, batch effects, DNA quality, and starting DNA quantity, we applied surrogate variable analysis (ARIC) and principal component analysis (MESA) to probe intensities of 43,316 Affymetrix autosomal SNPs [23]. We then calculated the residuals of a linear regression model with unadjusted mtDNA-CN as the dependent variable and age, sex, enrollment center, technical covariates, and white blood cell (WBC) count as independent variables in ARIC, and age, sex, collection center, race and principal components as independent variables in MESA. In ARIC, WBC count was missing in 14.9% of participants, and we imputed missing WBC as the study mean. In MESA, the rank-based inverse normal transformation was also performed to reduce the impact of outliers.

In CHS, mtDNA-CN was measured using multiplexed real-time quantitative polymerase chain reaction (qPCR) utilizing ABI TaqMan chemistry (Applied Biosystems) [12]. We calculated residuals using a linear mixed effect model stratified by race, with unadjusted mtDNA-CN as the dependent variable, and age, sex, collection site (fixed effects), and qPCR plate (random effect) as independent variables. The mtDNA-CN residuals were standardized within each study (mean of 0 and standard deviation of 1), and this measure was used as our estimate of mtDNA-CN. The normal distribution of mtDNA-CN residuals depended on the goodness of fit of the model. The residuals were standardized within each study (mean of 0 and standard deviation of 1), and this measure was used as our estimate of mtDNA-CN.

### Atrial fibrillation

In ARIC, AF cases were identified through December 31, 2014, from three sources: electrocardiograms (ECGs) performed during study visits, review of hospital discharge codes, and death certificates [24]. At each study exam, a supine 12-lead resting ECG was performed and transmitted to the ARIC ECG Reading Center (Epidemiological Cardiology Research Center, Wake Forest School of Medicine, Winston Salem, NC) for automatic coding with E Marquette 12-SL program (GE Marquette, Milwaukee, WI). AF or atrial flutter was detected automatically by a computer and confirmed by a cardiologist. Hospitalization information during follow-up was obtained through annual follow-up phone calls and surveillance of local hospitals. Trained abstractors collected hospital discharge codes. AF cases detected in the same hospitalization with open cardiac surgery were excluded. The validity of identifying AF from hospital discharge codes has been established in epidemiological studies [24, 25]. The presence of AF was identified if ICD-9-CM codes 427.31 (AF) or 427.32 (atrial flutter) were listed. Finally, AF was identified from death certificates if ICD-9 427.3 or ICD-10 I48 codes were listed as a cause of death. The AF date was determined as the date of the first ECG with AF (4%), the time of first hospital discharge with AF codes (96%), or when AF was listed as a cause of death (0.1%), whichever occurred first.

In MESA, AF cases were identified through December 31, 2014, from three sources: hospital discharge diagnosis codes, Medicare claims data, and study ECGs [26]. Hospitalization information during follow-up was obtained through phone calls every 9–12 months, and medical records and discharge diagnoses were obtained subsequently. Additionally, for participants enrolled in fee-for-service Medicare, AF diagnoses were identified from inpatient, outpatient, and physician claims. Study ECGs from visit 5 (2010–2012) were also used to identify incident AF.

In CHS, AF cases were identified through December 31, 2012, from three sources: annual ECGs at each study visit through 1999, discharge diagnoses for all hospitalizations (ICD-9-CM code 427.31 or 427.32), and for those enrolled in fee-for-service Medicare, from inpatient, outpatient, or physician claims in Medicare data [25]. The date of AF diagnosis was based on the date of the first ECG indicating AF, the time of the first hospital discharge with AF codes, or the time of the first qualifying outpatient or physician claim, whichever occurred first.

### Other covariates

The measurement of other covariates in the three cohorts has been described previously [19–21]. Age, sex, race/ethnicity, alcohol intake, smoking status, physical activity, and medication use were self-reported. Alcohol consumption was categorized into never, former, and current for ARIC and MESA and into non-current and current for CHS. Body mass index was calculated as weight (kg) divided by height (m) squared. Hypertension was defined as systolic blood pressure ≥ 140 mmHg, diastolic blood pressure ≥ 90 mmHg, or current use of anti-hypertension medication. Physical activity was assessed via a modified Baecke Questionnaire in ARIC (scales 1–5), a modified Minnesota Leisure Time Physical Activity Questionnaire in CHS (scales 1–4), and as the total amount of intentional moderate or vigorous exercise performed in a usual week in MESA (MET-min/week). Prevalent heart failure was defined by hospital records, physician diagnosis, or self-reported history of treatment.

Plasma total cholesterol, HDL cholesterol, fasting glucose, and creatinine were measured in each study are previously described [19–21]. N-terminal pro-brain natriuretic peptide (NT-proBNP) was measured at visits 2 and 4 using an electrochemiluminescent immunoassay in ARIC and at baseline using the Elecsys 2010 analyzer in MESA and CHS. Diabetes was defined as fasting glucose ≥ 126 mg/dL, non-fasting glucose ≥ 200 mg/dL, or use of glycemic control medication. Estimated glomerular filtration rate (eGFR) was calculated using the Chronic Kidney Disease Epidemiology Collaboration (CKD-EPI) equation ARIC and MESA [27], and Modification of Diet in Renal Disease (CKD-MDRD) equation in CHS [27]. Kidney disease was defined as eGFR < 60 mL/min/1.73 m^2^.

### Statistical analyses

Follow-up started from the baseline visit and continued until the development of AF, death, dropout, or through December 31, 2014, in ARIC and MESA or December 31, 2012, in CHS, whichever occurred first. mtDNA-CN was categorized into cohort-specific quintiles. We used a Cox proportional hazards model to estimate hazard ratios (HR) and 95% confidence intervals (CI) for the association between mtDNA-CN and incident AF in each cohort. HRs compared quintiles 1st to 4th with the 5th quintile (reference). Linear trends across quintiles were tested by including a variable containing the median mtDNA-CN level of each quintile in the models. We also modeled mtDNA-CN as a continuous variable and estimated the HR comparing the 10th to the 90th percentile of mtDNA-CN. In addition, to evaluate non-linear dose-response relationships between mtDNA-CN and incident AF, we modeled mtDNA-CN as restricted cubic splines with knots at the 5th, 35th, 65th, and 95th percentiles of its distribution. Finally, we tested for potential interactions by age, sex, race, smoking, alcohol intake, BMI, hypertension, diabetes, and kidney disease.

All analyses were conducted separately in each cohort, and cohort-specific HRs were combined using a fixed-effects meta-analysis approach. In each cohort, we used 4 multivariate models with progressive degrees of adjustment. Model 1 was adjusted for age, sex, and race enrollment center groups. Model 2 was further adjusted for body mass index, height, smoking, alcohol intake, and physical activity. Model 3 was further adjusted for total and HDL cholesterol, cholesterol medication, hypertension, diabetes, and prevalent HF. Model 4 was further adjusted for NT-proBNP. As additional analyses, we computed missing mtDNA-CN in ARIC using multiple imputation with chained equation (MICE) based on age, sex, race/center groups, BMI, height, physical activity, total cholesterol, HDL cholesterol, hyperlipidemia medication, eGFR, BNP, smoking, alcohol consumption, diabetes, hypertension, and HF. We used a series of 20 imputations to derive the additional estimates. We also conducted a post hoc analysis using the inverse probability of attrition weighting (IPAW) to account for potential informative death censoring [28–30]. Time-varying predicted probabilities of the remaining alive at a 6-month binned follow-up interval for each participant were calculated using the Cox proportional hazards model with death as the outcome and all variables in model 3 as independent variables. Attrition weights were calculated as the inverse of the predicted probability.

Statistical analyses were performed using Stata version 15 for ARIC and MESA study and Stata version 12 for the CHS study (StataCorp LP, College Station, TX). All *p* values were 2-sided, and statistical significance was declared at *p* < 0.05.

## Results

The study included 19,709 participants in total (10,149 from ARIC, 5781 from MESA, and 3779 from CHS). The mean (standard deviation) age of study participants was 57.2 (5.9), 62.3 (10.3), and 72.2 (5.3) for ARIC, MESA, and CHS, respectively (Table 1); 43.5% of the participants were men (ARIC 42.4%, MESA 47.8%, and CHS 39.9%), and 69.4% participants were Whites (ARIC 79.0%, MESA 42.6%, and CHS 84.7%). Participants with lower mtDNA-CN were more likely to be current smokers, to have higher NT-proBNP, and to have diabetes and prevalent CKD (Additional files: Tables S1-S3). Age, male gender, BMI, current smoking, diabetes, and NT-proBNP are the most strongly associated with the risk of incident AF (Table 2).

**Table 1:** Baseline characteristics of study participants

Characteristic	ARIC	MESA	CHS
*N*	10,149	5781	3779
Age (years)	57.2 (5.9)	62.3 (10.3)	72.2 (5.3)
Male	4307 (42.4)	2762 (47.8)	1506 (39.9)
Race			
White	8018 (79.0)	2464 (42.6)	3199 (84.7)
Black	2131 (21.0)	1387 (24.0)	580 (15.3)
Chinese-American	–	764 (13.2)	–
Hispanic	–	1166 (20.2)	–
Smoking			
Never	4020 (39.6)	2925 (50.6)	1766 (46.7)
Former	3814 (37.6)	2117 (36.6)	1535 (40.6)
Current	2315 (22.8)	739 (12.8)	478 (12.6)
Current drinker	5886 (58.0)	3221 (55.7)	1927 (51.0)
Body mass index (kg/m^2^)	28.0 (5.5)	28.2 (5.5)	26.7 (4.7)
Total cholesterol (mg/dL)	209.8 (39.5)	194.4 (35.7)	212.9 (38.6)
HDL cholesterol (mg/dL)	50.2 (17.1)	51.0 (14.9)	55.5 (15.8)
Triglycerides (mg/dL)	115.0 (82.0, 162.0)	112.0 (78.0, 162.0)	119.0 (91.0, 162.0)
Systolic BP (mmHg)	122.0 (19.1)	126.4 (21.7)	136.3 (21.6)
Diastolic BP (mmHg)	72.2 (10.3)	71.8 (10.3)	71.2 (11.2)
NT-proBNP (pg/mL)	52.2 (29.0, 91.7)	56.0 (24.9, 115.3)	96.8 (51.4, 186.5)
Hypertension	3560 (35.1)	2569 (44.4)	2134 (56.5)
Diabetes	1413 (13.9)	700 (12.1)	510 (13.5)
Prevalent HF	395 (3.9)	–	58 (1.5)
Prevalent CKD	192 (1.9)	776 (13.4)	721 (19.1)

Values are mean (SD), number (%), or median (IQR)

**Table 2:** Hazard ratios for AF associated with different risk factors

	ARIC	MESA	CHS
**mtDNA-CN**	**0.94 (0.89, 0.99)**	0.96 (0.89, 1.03)	0.97 (0.91,1.03)
**Age, years**	**1.65 (1.55, 1.77)**	**2.22 (1.99, 2.48)**	**1.57 (1.46,1.68)**
**Sex**			
Female	Reference (1)	Reference (1)	Reference (1)
Male	**1.29 (1.08, 1.55)**	**1.47 (1.16, 1.87)**	0.87 (0.72,1.05)
**Race**			
White	Reference (1)	Reference (1)	Reference (1)
Black	**0.58 (0.49, 0.69)**	**0.67 (0.54, 0.84)**	**0.65 (0.53, 0.79)**
Chinese-American	NA	**1.40 (1.03, 1.90)**	NA
Hispanic	NA	0.91 (0.70, 1.19)	NA
**BMI, kg/m**^**2**^	**1.31 (1.24, 1.39)**	**1.28 (1.17, 1.40)**	**1.09 (1.02,1.17)**
**Smoking**			
Never	Reference (1)	Reference (1)	Reference (1)
Former	1.09 (0.95, 1.25)	1.06 (0.89, 1.25)	1.10 (0.97,1.25)
Current	**1.74 (1.49, 2.02)**	**1.56 (1.20, 2.04)**	1.23 (0.99,1.52)
**Physical activity**	0.98 (0.93, 1.04)	0.99 (0.90, 1.08)	0.99 (0.93,1.05)
**Alcohol**			
Never	Reference (1)	Reference (1)	Reference (1)
Former	1.03 (0.87, 1.23)	1.21 (0.95, 1.55)	NA
Current	0.88 (0.76, 1.03)	1.15 (0.91, 1.44)	0.97 (0.87,1.07)
**Total cholesterol, mg/dL**	0.97 (0.92, 1.03)	1.05 (0.97, 1.14)	0.95 (0.89,1.02)
**HDL cholesterol, mg/dL**	0.98 (0.92, 1.05)	1.00 (0.91, 1.09)	1.05 (0.98,1.13)
**Lipid-lowering medication**			
No	Reference (1)	Reference (1)	Reference (1)
Yes	1.09 (0.87, 1.35)	1.11 (0.92, 1.34)	1.08 (0.82,1.42)
**Diabetes**			
No	Reference (1)	Reference (1)	Reference (1)
Yes	**1.36 (1.17, 1.59)**	1.20 (0.97, 1.48)	**1.25 (1.06,1.48)**
**Hypertension**			
No	Reference (1)	Reference (1)	Reference (1)
Yes	**1.41 (1.11, 1.79)**	1.04 (0.88, 1.23)	**1.32 (1.16,1.49)**
**eGFR, mL/min/1.73 m**^**2**^	**1.09 (1.02, 1.16)**	1.06 (0.97, 1.16)	1.01 (0.94,1.08)

Associated with 1 standard deviation increase of continuous risk factors. Model adjusted for all variables in the table

**Fig. 2:**
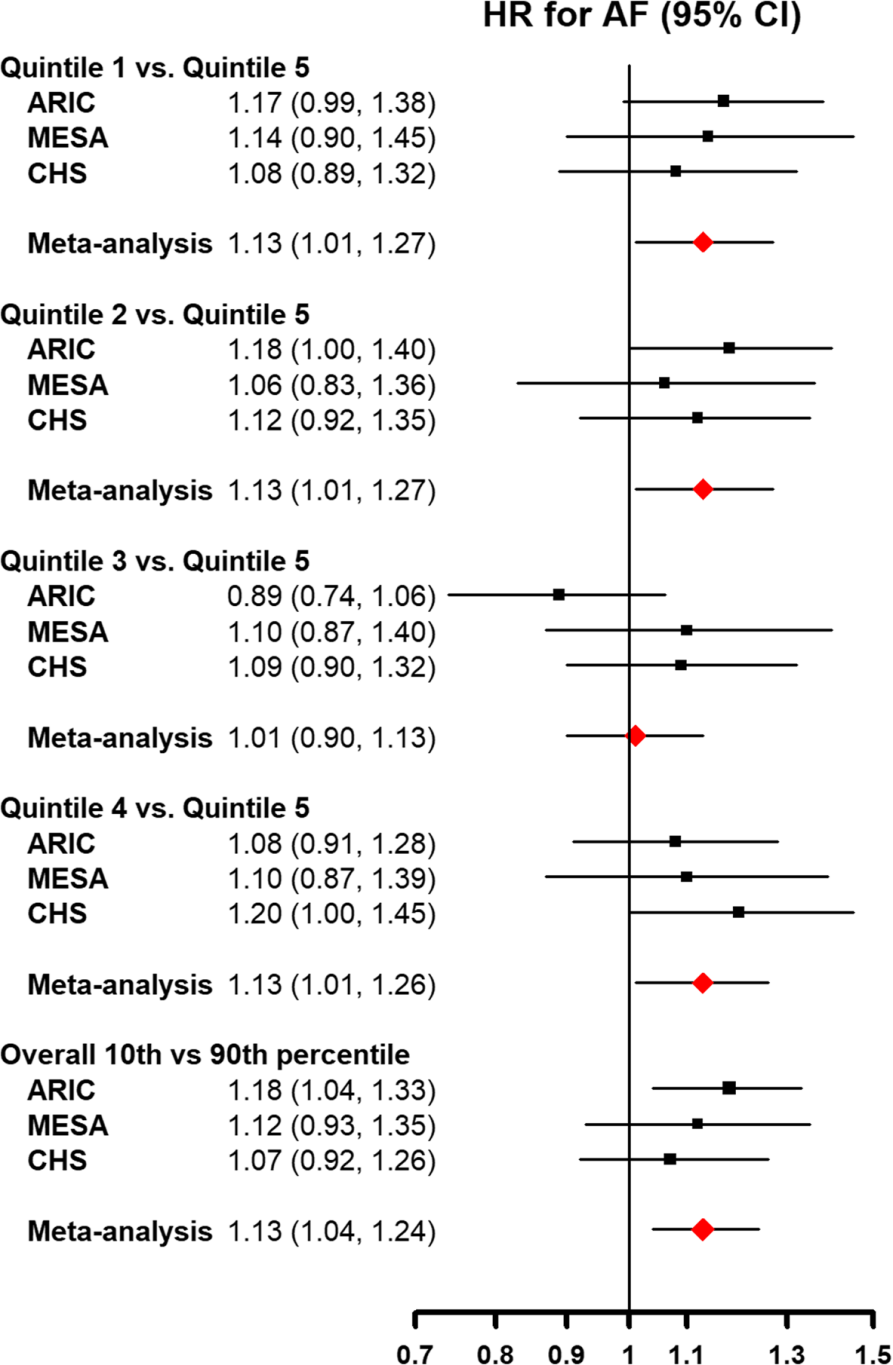
Hazard ratios for incident atrial fibrillation by levels of mtDNA copy number. The figure includes hazard ratios for comparing quintiles 1st to 4th with the 5th quintile (reference) of mtDNA copy number, as well as the hazard ratio for comparing the 10th to the 90th percentile of mtDNA copy number. Models were adjusted for age, sex, race/enrollment center, body mass index, height, smoking, alcohol intake, physical activity, total and HDL cholesterol, cholesterol medication, hypertension, diabetes, prevalent CHD, prevalent heart failure, eGFR, and log-transformed NT-proBNP at baseline

**Fig. 3:**
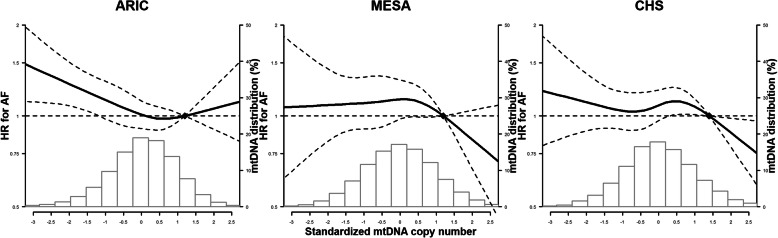
Spline regression analysis of incident atrial fibrillation by levels of mtDNA copy number. The curves represent adjusted hazard ratios (solid line) and their 95% confidence intervals (dashed lines) based on restricted cubic splines of mtDNA copy number with knots at the 5th, 35th, 65th, and 95th percentiles of its distribution. The reference value (diamond dot) was set at the 90th percentile of the distribution. Models were adjusted for age, sex, race/enrollment center, body mass index, height, smoking, alcohol intake, physical activity, total and HDL cholesterol, cholesterol medication, hypertension, diabetes, prevalent CHD, prevalent heart failure, eGFR, and log-transformed NT-proBNP at baseline. Histograms represent the frequency distribution of mtDNA copy number at baseline

The median follow-up time was 21.4 (IQR 14.8–23.3) years in ARIC, 12.9 (9.6–13.6) years in MESA, and 11.0 (5.9–17.1) years in CHS. During follow-up, 4021 participants developed incident AF (1761 in ARIC, 790 in MESA, and 1470 in CHS). The overall hazard ratios for incident AF for the 1st to 4th quintiles of mtDNA-CN compared to the 5th quintile are shown in Table 3. In the meta-analysis across the 3 cohorts using the fully adjusted model, participants in the 1st quintile of mitochondria DNA-CN had a 13% increased risk of incident AF compared to those in the 5th quintile (overall hazard ratio 1.13 [1.01, 1.27]) (Fig. 2). In models with mtDNA-CN as a continuous variable, participants at the 10th percentile of mtDNA-CN had a 13% increased risk of incident AF compared to those at the 90th percentile (overall hazard ratio 1.13 [1.04, 1.24]). Dose-response spline analysis also showed an inverse association between mtDNA-CN and AF for all three cohorts, with an approximately linear trend (the *p* values for non-linear spline terms were 0.20, 0.31, and 0.11 in ARIC, MESA, and CHS, respectively; Fig. 3).

**Table 3:** Hazard ratios for incident atrial fibrillation (AF) by quintiles of mtDNA copy number

	*N* events/*N* total	IR	Model 1*	Model 2^†^	Model 3^‡^	Model 4^§^
ARIC						
mtDNA-CN quintiles						
First	399/2029	10.9	**1.26 (1.09, 1.46)**	**1.21 (1.05, 1.40)**	**1.20 (1.04, 1.39)**	1.17 (0.99, 1.38)
Second	355/2035	9.5	1.09 (0.94, 1.27)	1.11 (0.95, 1.29)	1.10 (0.94, 1.27)	**1.18 (1.00, 1.40)**
Third	319/2037	8.5	0.97 (0.83, 1.13)	0.93 (0.80, 1.09)	0.94 (0.80, 1.09)	0.89 (0.74, 1.06)
Fourth	356/2031	9.3	1.07 (0.92, 1.24)	1.08 (0.93, 1.25)	1.08 (0.93, 1.25)	1.08 (0.91, 1.28)
Fifth	332/2017	8.9	Reference	Reference	Reference	Reference
*p*-trend			0.002	0.008	0.01	0.03
10th vs 90th percentile	1761/10,149	9.4	**1.20 (1.07, 1.34)**	**1.18 (1.05, 1.31)**	**1.16 (1.04, 1.30)**	**1.18 (1.04, 1.33)**
MESA						
mtDNA-CN quintiles						
First	157/1154	12.4	1.04 (0.83, 1.30)	1.02 (0.82, 1.28)	1.01 (0.81, 1.26)	1.14 (0.90, 1.45)
Second	150/1154	11.6	1.01 (0.81, 1.27)	0.99 (0.79, 1.24)	0.97 (0.78, 1.22)	1.06 (0.83, 1.36)
Third	161/1166	12.3	1.04 (0.83, 1.30)	1.02 (0.82, 1.28)	1.02 (0.82, 1.27)	1.10 (0.87, 1.40)
Fourth	169/1159	13.1	1.08 (0.87, 1.34)	1.05 (0.84, 1.31)	1.04 (0.83, 1.29)	1.10 (0.87, 1.39)
Fifth	153/1148	11.8	Reference	Reference	Reference	Reference
*p*-trend			0.91	0.98	0.88	0.37
10th vs 90th percentile	790/5781	12.2	1.05 (0.88, 1.25)	1.03 (0.87, 1.23)	1.03 (0.86, 1.23)	1.12 (0.93, 1.35)
CHS						
mtDNA-CN quintiles						
First	283/755	32.0	1.16 (0.98,1.38)	1.16 (0.98,1.37)	1.11 (0.93,1.31)	1.08 (0.89,1.32)
Second	292/756	31.9	1.12 (0.95,1.32)	1.12 (0.95,1.32)	1.09 (0.92,1.28)	1.12 (0.92,1.35)
Third	296/756	31.8	1.10 (0.94,1.30)	1.10 (0.94,1.30)	1.08 (0.92,1.28)	1.09 (0.90,1.32)
Fourth	317/756	33.6	**1.19 (1.01,1.39)**	**1.18 (1.00,1.39)**	1.15 (0.98,1.35)	**1.20 (1.00,1.45)**
Fifth	282/756	29.0	Reference	Reference	Reference	Reference
*p*-trend			0.16	0.18	0.38	0.65
10th vs 90th percentile	1470/3779	31.6	1.12 (0.98,1.27)	1.11 (0.97,1.27)	1.07 (0.94,1.23)	1.07 (0.92,1.26)
Meta-analysis						
mtDNA-CN quintiles						
First	839/3938	14.4	**1.18 (1.07, 1.30)**	**1.15 (1.05, 1.27)**	**1.13 (1.02, 1.25)**	**1.13 (1.01, 1.27)**
Second	797/3945	13.4	1.09 (0.98, 1.20)	1.09 (0.99, 1.20)	**1.07 (0.97, 1.18)**	**1.13 (1.01, 1.27)**
Third	776/3959	12.9	1.03 (0.93, 1.14)	1.01 (0.92, 1.21)	1.00 (0.91, 1.11)	1.01 (0.90, 1.13)
Fourth	842/3946	13.9	**1.12 (1.01, 1.23)**	**1.11 (1.00, 1.22)**	1.10 (0.99, 1.21)	**1.13 (1.01, 1.26)**
Fifth	767/3921	12.8	Reference	Reference	Reference	Reference
*p*-trend			0.006	0.01	0.03	0.03
10th vs 90th percentile	4021/19,709	13.5	**1.14 (1.06, 1.23)**	**1.13 (1.04, 1.22)**	**1.11 (1.03, 1.20)**	**1.13 (1.04, 1.24)**

*IR* incidence rate (per 1000 person-years)

*Model 1: adjusted for age, sex, and race/enrollment center groups

^†^Model 2: further adjusted for body mass index, height, smoking, alcohol intake, and physical activity

^‡^Model 3: further adjusted for total and HDL cholesterol, cholesterol medication, hypertension, diabetes, prevalent heart failure, and eGFR at baseline

^§^Model 4: further adjusted for log-transformed NT-proBNP

In the stratified analysis, there was no evidence of interaction for the associations between mtDNA-CN and AF across all subgroups evaluated (Additional Files: Figure S1), except that they were stronger in hypertensive than in non-hypertensive participants from ARIC (*p* interaction = 0.03). Sensitivity analysis excluding non-Black and non-White participants in MESA, as well as additional analyses using MICE for missing mtDNA-CN in ARIC and IPAW to account for informative censoring in all cohorts, showed similar results (Additional Files: Table S4-S6).

## Discussion

In three large population-based prospective cohort studies, mtDNA-CN was inversely associated with the risk of incident AF, independent of traditional risk factors. The association was not statistically different across race and sex groups. This novel association indicates a potential role of mitochondrial dysfunction in atrial arrhythmias and adds to the pathophysiological evidence from basic science studies supporting the role of mitochondrial mechanisms in the genesis of AF.

Animal models and molecular studies suggest that mitochondrial dysfunction is associated with adverse CVD outcomes and subclinical atherosclerosis [31, 32], and some of these associations have been confirmed in population-based studies. However, previous studies on mtDNA-CN and other CVD outcomes reported their results using different metrics, and the magnitude of reported estimates was incomparable. In ARIC, MESA, and CHS, low levels of mtDNA-CN were associated with increased risk of incident CVD (HR for a 1-SD decrease in mtDNA-CN, 1.29 [95% CI, 1.24–1.33]), coronary heart disease (HR for a 1-SD decrease in mtDNA-CN, 1.29 [1.24–1.33]), sudden cardiac death (HR comparing the 1st to the 5th quintiles of mtDNA-CN, 2.24 [1.58–3.19]), and all-cause mortality (HR comparing the 1st to the 5th quintiles of mtDNA-CN, 1.47 [95% CI, 1.33–1.62]) [10–12]. Low levels of mtDNA-CN were also associated with CVD risk factors, including hypertension, diabetes, and chronic kidney disease [14–16]. Since clinical CVD events and CVD risk factors are also risk factors for AF, the inverse associations between mtDNA-CN and AF could also be mediated through the traditional CVD pathways. As participants in our analysis were free of CHD at baseline and we adjusted for CVD risk factors, we identified an independent and statistically significant association between mtDNA-CN and incident AF after accounting for CVD and traditional risk factors. Furthermore, since low mtDNA-CN levels preceded the development of AF and other CVD events in ARIC, CHS, and MESA, maintaining CVD health may require adequate cell energy mechanisms and mitochondrial function for preserved cardiac contractility, electrical activity, and endothelial function.

AF is triggered by structural and electrophysiological remodeling changes in the atrial myocardium, which in turn adversely affect cardiac function and increase the risk of mortality, stroke, and peripheral embolism [33]. Risk factors of AF include older age, male sex, and the presence of clinical cardiomyopathy, coronary artery disease, and traditional CVD risk factors (hypertension, diabetes, obesity, and smoking) [25, 34, 35]. Our results suggest that mtDNA-CN may be a novel risk factor for AF. As low levels of mtDNA-CN are a marker for mitochondrial dysfunction and abnormal ATP production, our findings suggest that myocyte electrical activity could be compromised by mitochondrial dysfunction and insufficient energy supply.

The mechanisms underlying the association between mtDNA-CN and AF are unknown, but previous mechanistic research and in vitro studies provide some leads. Most of the energy for cardiomyocyte electrical activity and cardiac muscle contraction is supplied by the mitochondria through the oxidative phosphorylation pathway [18]. The generation of cellular reactive oxygen species (ROS) associated with mitochondrial dysfunction could increase arrhythmia susceptibility by affecting energy-dissipating ion channels and transporters, including the proteins involved in excitation-contraction coupling. Indeed, disrupted intracellular Ca^2+^ homeostasis has been shown to contribute to the pathogenesis of AF [17, 18, 36]. Increased ROS can also impair gap junction regulation and affect voltage-gated sodium-potassium channels, which increases electrical heterogeneity and causes early afterdepolarizations [18].

Apart from ROS, mitochondrial dysfunction impairs ATP synthesis, affecting cardiomyocyte energy metabolism, sarcolemmal and sarcoplasmic ion channel function, intracellular cation homeostasis, and membrane excitability, which are essential in maintaining the electrical activity of cardiac cells [36]. The reduced energy production promotes the opening of sarcolemmal K_ATP_ channels, which has arrhythmogenic effects by conferring shortened action potential duration and excitation wavelengths [37].

Some limitations of this study need to be considered. Differences in measurement methods in mtDNA-CN, AF, and covariates may contribute to the heterogeneity of the results across the 3 cohorts. Reassuringly, the direction of the association of mtDNA-CN and incident AF was consistent in ARIC, CHS, and MESA, adding weight to the validity of our findings. mtDNA-CN was collected from the peripheral blood and was thus not a direct measurement of mitochondrial function in atrial myocytes. However, mtDNA from the peripheral blood has been shown to be strongly correlated with mtDNA from cardiomyocyte (coefficient of correlation > 0.5) [13, 38]. Therefore, the mtDNA-CN from the peripheral blood could be used as a marker for myocardial mitochondrial function. Furthermore, mtDNA-CN was measured only at a single time point, and variability due to changes over time was not captured. Finally, we could not evaluate the association between mtDNA-CN with various subtypes of AF. Since AF was obtained primarily based on hospital discharge codes in all 3 studies, the diagnoses were mostly persistent or permanent forms of AF, whereas asymptomatic paroxysmal AF, the most common type of AF, is often undetected. Future studies with repeated measures of mtDNA markers may provide a better evaluation of longitudinal changes in mtDNA function and its impact on AF risk.

The strengths of this study include the prospective design, the long follow-up, the rigorous quality control procedures of the individual cohorts, the large sample size, and the heterogeneous composition of the study population, including men and women from multiple race/ethnicity groups and a wide age range from middle-aged adults to elderly participants.

## Conclusion

In three prospective community-based cohorts, mtDNA-CN levels in the peripheral blood were inversely associated with the risk of AF independent of traditional risk factors. These findings implicate mtDNA-CN as a novel risk factor for AF. Further research is warranted to better understand the underlying mechanisms, to better characterize the dose-response shape of the association, and to evaluate the role of mtDNA-CN in the prevention and management of AF risk.

## Supplementary information

**Additional file 1: Figure S1**. Hazard ratios for incident atrial fibrillation by levels of mtDNA copy number in pre-specified subgroups. Age groups were ≤65 and >65 for ARIC and MESA, ≤70 and >70 for CHS. Hazard ratios are for comparing the 10th to the 90th percentile of mtDNA copy number. Models were adjusted for age, sex, race/enrollment center, body mass index, height, smoking, alcohol intake, physical activity, total and HDL cholesterol, cholesterol medication, hypertension, diabetes, prevalent CHD, prevalent heart failure, eGFR and log-transformed NT-proBNP at baseline. **Table S1.** Baseline characteristics of study participants by mtDNA-CN quintiles in ARIC. **Table S2.** Baseline characteristics of study participants by mtDNA-CN quintiles in MESA. **Table S3.** Baseline characteristics of study participants by mtDNA-CN quintiles in CHS. **Table S4.** Hazard ratios for incident AF by quintiles of mtDNA copy number, among Black and White MESA participants. **Table S5.** Hazard ratios for incident AF by quintiles of mtDNA copy number in ARIC, using multiple imputation by chained equations (MICE) to impute for missing mtDNA-CN. **Table S6.** Hazard ratios for incident AF by quintiles of mtDNA copy number, using inverse probability weighting to account for death as dependent censoring.

## Data Availability

The data that support the findings of this study are available from the ARIC, MESA, and CHS repository but are not publicly available. Restrictions apply to the availability of these data, which were used under license for the current study.

## Abbreviations

ARIC: Atherosclerosis Risk in Communities Study
ATP: Adenosine triphosphate
AF: Atrial fibrillation
CVD: Cardiovascular disease
CHS: Cardiovascular Health Study
DNA: Deoxyribonucleic acid
MESA: Multi-Ethnic Study of Atherosclerosis
mtDNA-CN: Mitochondria deoxyribonucleic acid copy number
ROS: Reactive oxygen species
SNP: Single nucleotide polymorphisms
