# Supplementary material for: Mitochondrial DNA copy number and incident atrial fibrillation
Source: BMC Med. 2020 Sep 16;18:246. doi: 10.1186/s12916-020-01715-6 (PMC7493408; doi:10.1186/s12916-020-01715-6)

**Table S1.**

| Characteristic | First | Second | Third | Fourth | Fifth | P-value |
| --- | --- | --- | --- | --- | --- | --- |
| N | 2029 | 2035 | 2037 | 2031 | 2017 |  |
| Age (year) | 57.2 (6.0) | 57.1 (6.0) | 57.3 (5.9) | 57.0 (5.9) | 57.3 (5.8) | 0.54 |
| Male | 849 (41.8) | 861 (42.3) | 863 (42.4) | 847 (41.7) | 887 (44.0) | 0.61 |
| Race |  |  |  |  |  | 0.48 |
| White | 1628 (80.2) | 1612 (79.2) | 1606 (78.8) | 1582 (77.9) | 1590 (78.8) |  |
| Black | 401 (19.8) | 423 (20.8) | 431 (21.2) | 449 (22.1) | 427 (21.2) |  |
| Smoking |  |  |  |  |  | 0.002 |
| Never | 764 (37.7) | 841 (41.3) | 806 (39.6) | 812 (40.0) | 797 (39.5) |  |
| Former | 733 (36.1) | 727 (35.7) | 775 (38.0) | 784 (38.6) | 795 (39.4) |  |
| Current | 532 (26.2) | 467 (22.9) | 456 (22.4) | 435 (21.4) | 425 (21.1) |  |
| Current alcohol drinker | 1125 (55.4) | 1202 (59.1) | 1168 (57.3) | 1228 (60.5) | 1163 (57.7) | 0.02 |
| Physical activity | 2.4 (0.8) | 2.5 (0.8) | 2.5 (0.8) | 2.5 (0.8) | 2.5 (0.8) | 0.38 |
| Body mass index (kg/m^2^) | 27.9 (5.6) | 27.7 (5.2) | 28.2 (5.6) | 27.8 (5.4) | 28.0 (5.5) | 0.04 |
| Total cholesterol (mg/dL) | 209.3 (41.1) | 208.7 (39.2) | 210.0 (37.9) | 209.8 (39.0) | 211.1 (40.3) | 0.39 |
| HDL cholesterol (mg/dL) | 48.9 (16.8) | 50.1 (17.2) | 50.3 (16.6) | 51.5 (17.8) | 50.1 (17.1) | <0.001 |
| Triglycerides (mg/dL) | 116.0 (83.0, 162.0) | 115.0 (82.0, 161.0) | 116.0 (82.0, 165.0) | 109.0 (81.0, 158.0) | 118.0 (85.0, 162.0) | 0.01 |
| Systolic BP (mm Hg) | 122.7 (19.6) | 122.0 (19.7) | 122.1 (18.8) | 121.5 (18.8) | 121.7 (18.5) | 0.33 |
| Diastolic BP (mm Hg) | 72.3 (10.5) | 72.2 (10.4) | 72.1 (10.1) | 72.3 (9.9) | 72.3 (10.5) | 0.97 |
| NT-proBNP (pg/mL) | 56.5 (31.5, 102.0) | 51.3 (29.4, 92.8) | 52.3 (28.0, 90.9) | 50.5 (28.7, 86.9) | 50.9 (27.6, 86.2) | <0.001 |
| Hypertension | 748 (36.9) | 716 (35.2) | 695 (34.1) | 700 (34.5) | 701 (34.8) | 0.39 |
| Diabetes | 336 (16.6) | 296 (14.5) | 269 (13.2) | 245 (12.1) | 267 (13.2) | <0.001 |
| Prevalent HF | 73 (3.6) | 73 (3.6) | 80 (3.9) | 76 (3.7) | 93 (4.6) | 0.42 |
| Prevalent CKD | 53 (2.6) | 38 (1.9) | 34 (1.7) | 33 (1.6) | 34 (1.7) | 0.11 |

Values the Table are mean (SD), number (%), or median (IQR)

**Table S2.**

| Characteristic | First | Second | Third | Fourth | Fifth | P-value |
| --- | --- | --- | --- | --- | --- | --- |
| N | 1,154 | 1,154 | 1,166 | 1,159 | 1,148 |  |
| Age (year) | 62.5 (10.4) | 62.0 (10.3) | 62.2 (10.1) | 62.5 (10.4) | 62.4 (10.1) | 0.78 |
| Male | 540 (46.8) | 556 (48.2) | 573 (49.1) | 547 (47.2) | 546 (47.6) | 0.81 |
| Race / enrollment center |  |  |  |  |  | 0.10 |
| White | 492 (42.6) | 511 (44.3) | 486 (41.7) | 493 (42.5) | 482 (42.0) |  |
| Black | 140 (12.1) | 147 (12.7) | 167 (14.3) | 172 (14.8) | 138 (12.0) |  |
| Chinese-American | 303 (26.3) | 261 (22.6) | 254 (21.8) | 270 (23.3) | 299 (26.0) |  |
| Hispanic | 219 (19.0) | 235 (20.4) | 259 (22.2) | 224 (19.3) | 229 (19.9) |  |
| Smoking |  |  |  |  |  | 0.42 |
| Never | 585 (50.7) | 595 (51.6) | 594 (50.9) | 582 (50.2) | 569 (49.6) |  |
| Former | 410 (35.5) | 401 (34.7) | 424 (36.4) | 432 (37.3) | 450 (39.2) |  |
| Current | 159 (13.8) | 158 (13.7) | 148 (12.7) | 145 (12.5) | 129 (11.2) |  |
| Current alcohol drinker | 649 (56.2) | 642 (55.6) | 646 (55.4) | 637 (55.0) | 647 (56.4) | 0.96 |
| Physical activity | 5638.3 (6338.1) | 5510.9 (5500.7) | 6056.2 (6305.4) | 5404.8 (5591.3) | 5677.2 (5381.9) | 0.04 |
| Body mass index (kg/m^2^) | 28.4 (5.8) | 28.2 (5.4) | 28.2 (5.5) | 28.2 (5.4) | 27.9 (5.2) | 0.2 |
| Total cholesterol (mg/dL) | 194.5 (36.4) | 193.7 (37.0) | 195.0 (35.5) | 193.9 (33.1) | 195.0 (36.3) | 0.84 |
| HDL cholesterol (mg/dL) | 50.9 (14.6) | 50.4 (15.0) | 50.0 (14.1) | 51.4 (15.2) | 52.2 (15.3) | 0.004 |
| Triglycerides (mg/dL) | 114.0 (79.0,  166.0) | 113.5 (79.0,  162.0) | 116.5 (79.0,  170.0) | 110.0 (78.0,  159.0) | 106.0 (77.5,  156.0) | 0.08 |
| Systolic BP (mm Hg) | 127.8 (20.8) | 127.0 (21.8) | 125.9 (21.3) | 126.2 (22.1) | 125.0 (22.3) | 0.02 |
| Diastolic BP (mm Hg) | 72.1 (10.3) | 72.4 (10.2) | 71.7 (10.3) | 71.3 (10.4) | 71.3 (10.1) | 0.03 |
| NT-proBNP (pg/mL) | 57.3 (23.8 - 116.3) | 52.4 (25.2 - 110.0) | 51.7 (23.3 - 108.5) | 59.1 (26.8 - 120.9) | 59.8 (26.3 - 120.9) | 0.14 |
| Hypertension | 551 (47.7) | 518 (44.9) | 504 (43.2) | 510 (44.0) | 486 (42.3) | 0.09 |
| Diabetes | 159 (13.8) | 147 (12.7) | 138 (11.8) | 135 (11.6) | 121 (10.5) | 0.17 |
| Prevalent CKD | 185 (16.0) | 150 (13.0) | 162 (13.9) | 139 (12.0) | 140 (12.2) | 0.03 |

Values the Table are mean (SD), number (%), or median (IQR)

**Table S3.**

| Characteristic | First | Second | Third | Fourth | Fifth | p-value |
| --- | --- | --- | --- | --- | --- | --- |
| N | 755 | 756 | 756 | 756 | 756 |  |
| Age (year) | 71.9 (5.2) | 72.3 (5.3) | 72.2 (5.3) | 72.5 (5.4) | 72.1 (5.2) | 0.20 |
| Male gender | 290 (38.4) | 302 (39.9) | 325 (43.0) | 280 (37.0) | 309 (40.9) | 0.16 |
| Race |  |  |  |  |  | 0.71 |
| White | 638 (84.5) | 642 (84.9) | 651 (86.1) | 632 (83.6) | 636 (84.1) |  |
| Black | 117 (15.5) | 114 (15.1) | 105 (13.9) | 124 (16.4) | 120 (15.9) |  |
| Smoking |  |  |  |  |  | 0.51 |
| Never | 348 (46.1) | 360 (47.6) | 359 (47.5) | 350 (46.3) | 349 (46.2) |  |
| Former | 298 (39.5) | 294 (38.9) | 303 (40.1) | 314 (41.5) | 326 (43.1) |  |
| Current | 109 (14.4) | 102 (13.5) | 94 (12.4) | 92 (12.2) | 81 (10.7) |  |
| Current alcohol drinker | 344 (45.6) | 379 (50.1) | 389 (51.5) | 401 (53.0) | 414 (54.8) | 0.005 |
| Physical activity | 2.2 (1.0) | 2.2 (1.0) | 2.3 (1.0) | 2.3 (1.0) | 2.4 (1.0) | 0.001 |
| Body mass index (kg/m^2^) | 26.7 (4.9) | 26.5 (4.9) | 26.7 (4.5) | 26.7 (4.7) | 26.7 (4.6) | 0.94 |
| Total cholesterol (mg/dL) | 210.6 (39.0) | 211.7 (37.4) | 213.3 (38.0) | 213.7 (39.8) | 215.1 (38.8) | 0.17 |
| HDL cholesterol (mg/dL) | 54.4 (15.5) | 56.0 (16.5) | 55.2 (15.6) | 55.7 (15.4) | 56.1 (16.0) | 0.23 |
| Triglycerides (mg/dL) | 122.0 (93.0,  170.0) | 116.0 (93.5, 155.0) | 117.0 (90.0, 159.5) | 119.0 (90.0, 164.5) | 120.0 (90.0, 160.0) | 0.45 |
| Systolic BP (mm Hg) | 135.9 (21.8) | 136.8 (22.2) | 137.2 (21.6) | 136.1 (21.1) | 135.4 (21.4) | 0.48 |
| Diastolic BP (mm Hg) | 70.6 (11.3) | 71.5 (11.9) | 72.2 (11.1) | 70.8 (11.0) | 70.8 (10.7) | 0.04 |
| NT-proBNP (pg/mL) | 102.0 (52.5 ,193.8) | 110.6 (59.7 ,203.8) | 96.2 (51.5 ,185.0) | 95.0 (50.5 ,184.8) | 86.6 (45.7 ,156.6) | 0.001 |
| Hypertension | 424 (56.2) | 433 (57.3) | 442 (58.5) | 432 (57.1) | 403 (53.3) | 0.32 |
| Diabetes | 120 (15.9) | 106 (14.0) | 79 (10.4) | 102 (13.5) | 103 (13.6) | 0.04 |
| Prevalent HF | 18 (2.4) | 12 (1.6) | 8 (1.1) | 14 (1.9) | 6 (0.8) | 0.09 |
| Prevalent CKD | 187 (24.8) | 148 (19.6) | 161 (21.3) | 121 (16.0) | 104 (13.8) | <0.001 |

Values the Table are mean (SD), number (%), or median (IQR)

**Table S4.**

| mtDNA-CN quintiles | N events / N total | IR | Model 1* | Model 2† | Model 3‡ | Model 4§ |
| --- | --- | --- | --- | --- | --- | --- |
| First | 109 / 795 | 12.6 | 1.04 (0.80, 1.36) | 1.03 (0.78, 1.34) | 1.01 (0.77, 1.33) | 1.16 (0.87, 1.56) |
| Second | 92 / 772 | 10.5 | 0.91 (0.69, 1.21) | 0.88 (0.67, 1.17) | 0.87 (0.66, 1.16) | 0.98 (0.72, 1.34) |
| Third | 112 / 740 | 13.3 | 1.14 (0.87, 1.49) | 1.11 (0.85, 1.46) | 1.10 (0.84, 1.44) | 1.26 (0.93, 1.69) |
| Fourth | 128 / 763 | 15.0 | 1.25 (0.96, 1.61) | 1.21 (0.93, 1.56) | 1.18 (0.91, 1.53) | 1.27 (0.95, 1.68) |
| Fifth | 105 / 781 | 11.7 | Reference | Reference | Reference | Reference |
| p-trend |  |  | 0.56 | 0.49 | 0.45 | 0.83 |
| 10^th^ vs 90^th^ percentile | 546 / 3,851 | 12.6 | 0.99 (0.80, 1.22) | 0.97 (0.78, 1.20) | 0.96 (0.78, 1.20) | 1.07 (0.85, 1.34) |

* Model 1: Adjusted for age, sex and race/enrollment center groups.
† Model 2: Further adjusted for + body mass index, height, smoking, alcohol intake, and physical activity.
‡ Model 3: Further adjusted for total and HDL cholesterol, cholesterol medication, hypertension, diabetes, prevalent heart failure, and eGFR at baseline.

§ Model 4: Further adjusted for log-transformed NT-proBNP.

Abbreviations: IR: incidence rate (per 1,000 person-years)

**Table S5.**

| ARIC |  |  |  |  |
| --- | --- | --- | --- | --- |
|  | Model 1* | Model 2† | Model 3‡ | Model 4§ |
| mtDNA-CN quintiles |  |  |  |  |
| First | 1.24 (1.08, 1.43) | 1.20 (1.04, 1.37) | 1.18 (1.03, 1.35) | 1.14 (0.99, 1.31) |
| Second | 1.10 (0.96, 1.27) | 1.10 (0.96, 1.27) | 1.10 (0.96, 1.26) | 1.09 (0.94, 1.25) |
| Third | 0.98 (0.84, 1.14) | 0.95 (0.81, 1.11) | 0.95 (0.81, 1.11) | 0.92 (0.78, 1.08) |
| Fourth | 1.04 (0.90, 1.21) | 1.04 (0.90, 1.21) | 1.05 (0.90, 1.22) | 1.04 (0.90, 1.21) |
| Fifth | Reference | Reference | Reference | Reference |
| p-trend | <0.001 | 0.002 | 0.003 | 0.01 |
| 10^th^ vs 90^th^ percentile | 1.21 (1.09, 1.35) | 1.18 (1.06, 1.32) | 1.16 (1.04, 1.29) | 1.13 (1.01, 1.26) |

* Model 1: Adjusted for age, sex and race/enrollment center groups.
† Model 2: Further adjusted for + body mass index, height, smoking, alcohol intake, and physical activity.
‡ Model 3: Further adjusted for total and HDL cholesterol, cholesterol medication, hypertension, diabetes, prevalent heart failure, and eGFR at baseline.

§ Model 4: Further adjusted for log-transformed NT-proBNP.

**Table S6.**

| ARIC |  |  |  |  |
| --- | --- | --- | --- | --- |
|  | Model 1* | Model 2† | Model 3‡ | Model 4§ |
| mtDNA-CN quintiles |  |  |  |  |
| First | 1.30 (1.10, 1.54) | 1.24 (1.05, 1.46) | 1.22 (1.04, 1.45) | 1.19 (0.99, 1.44) |
| Second | 1.10 (0.94, 1.30) | 1.11 (0.94, 1.31) | 1.11 (0.94, 1.31) | 1.24 (1.03, 1.49) |
| Third | 1.03 (0.85, 1.25) | 0.99 (0.82, 1.20) | 0.99 (0.82, 1.20) | 1.01 (0.80, 1.26) |
| Fourth | 1.12 (0.95, 1.32) | 1.13 (0.95, 1.33) | 1.12 (0.95, 1.32) | 1.14 (0.94, 1.37) |
| Fifth | Reference | Reference | Reference | Reference |
| p-trend | 0.004 | 0.02 | 0.03 | 0.05 |
| 10^th^ vs 90^th^ percentile | 1.23 (1.08, 1.40) | 1.20 (1.05, 1.36) | 1.18 (1.04, 1.35) | 1.20 (1.04, 1.38) |
| MESA |  |  |  |  |
|  | Model 1* | Model 2† | Model 3‡ | Model 4§ |
| mtDNA-CN quintiles |  |  |  |  |
| First | 1.07 (0.84, 1.36) | 1.05 (0.82, 1.33) | 1.03 (0.81, 1.30) | 1.18 (0.91, 1.51) |
| Second | 1.00 (0.79, 1.26) | 0.98 (0.77, 1.23) | 0.96 (0.76, 1.21) | 1.05 (0.81, 1.34) |
| Third | 1.01 (0.81, 1.28) | 1.00 (0.80, 1.27) | 1.00 (0.80, 1.26) | 1.08 (0.84, 1.38) |
| Fourth | 1.08 (0.86, 1.35) | 1.04 (0.83, 1.31) | 1.03 (0.82, 1.29) | 1.07 (0.84, 1.36) |
| Fifth | Reference | Reference | Reference | Reference |
| p-trend | 0.79 | 0.89 | 0.99 | 0.26 |
| 10^th^ vs 90^th^ percentile | 1.06 (0.88, 1.27) | 1.04 (0.86, 1.26) | 1.03 (0.86, 1.25) | 1.13 (0.93, 1.38) |
| CHS |  |  |  |  |
|  | Model 1* | Model 2† | Model 3‡ | Model 4§ |
| mtDNA-CN quintiles |  |  |  |  |
| First | 1.04 (0.83,1.31) | 1.04 (0.83,1.31) | 1.00 (0.80,1.26) | 1.06 (0.84,1.33) |
| Second | 1.15 (0.93,1.41) | 1.15 (0.93,1.41) | 1.11 (0.90,1.37) | 1.17 (0.94,1.44) |
| Third | 1.08 (0.88,1.34) | 1.09 (0.88,1.34) | 1.05 (0.85,1.29) | 1.05 (0.85,1.29) |
| Fourth | 1.21 (0.99,1.47) | 1.20 (0.99,1.47) | 1.17 (0.96,1.43) | 1.26 (1.03,1.54) |
| Fifth | Reference | Reference | Reference | Reference |
| p-trend | 0.88 | 0.88 | 0.85 | 0.96 |
| 10^th^ vs 90^th^ percentile | 1.02 (0.86,1.22) | 1.02 (0.86,1.22) | 0.98 (0.83,1.17) | 1.04 (0.88,1.24) |

* Model 1: Adjusted for age, sex and race/enrollment center groups.
† Model 2: Further adjusted for + body mass index, height, smoking, alcohol intake, and physical activity.
‡ Model 3: Further adjusted for total and HDL cholesterol, cholesterol medication, hypertension, diabetes, prevalent heart failure, and eGFR at baseline.

§ Model 4: Further adjusted for log-transformed NT-proBNP.

**Figure S1.**


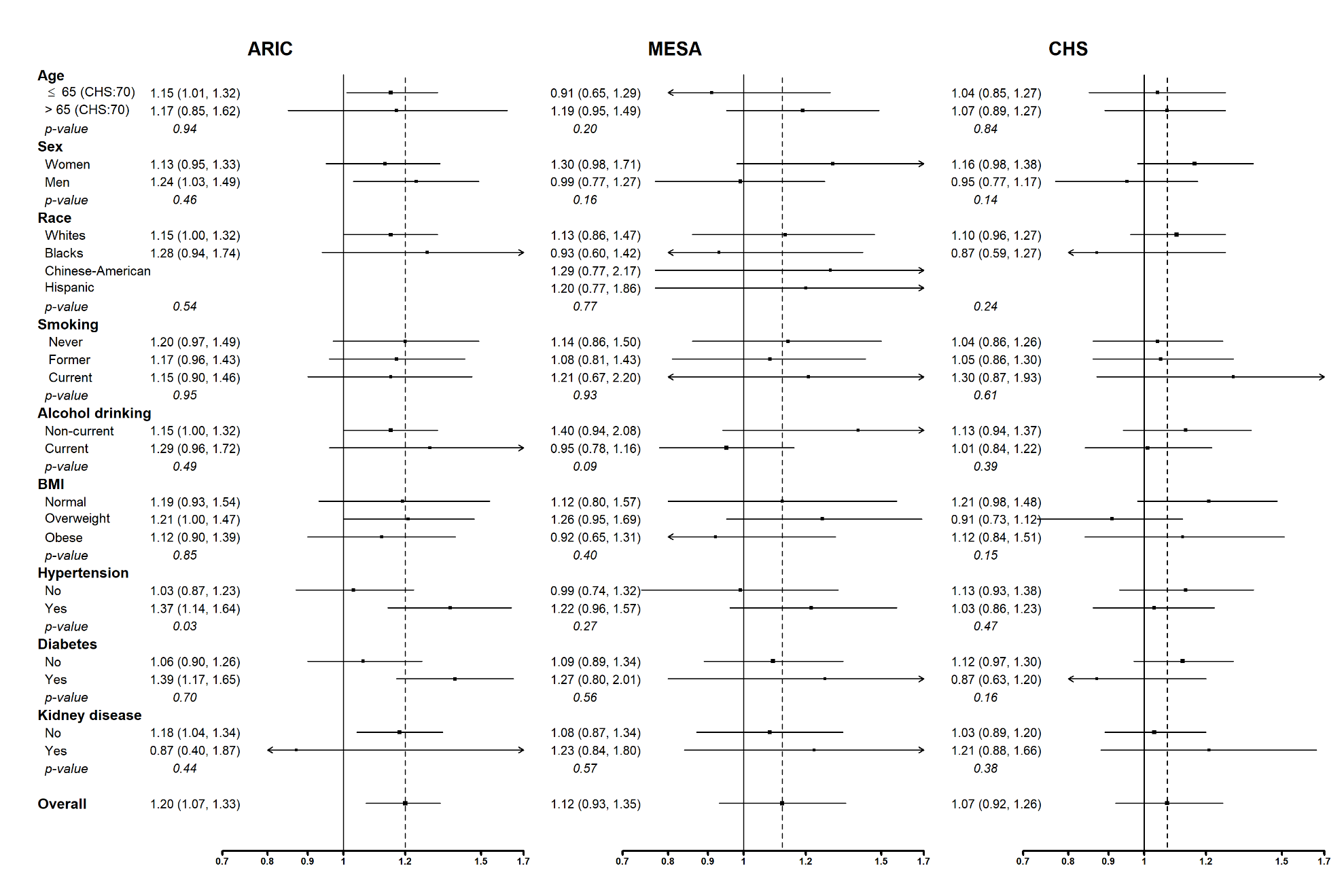

Supplement: Supplementary file 1 — Additional file 1: Figure S1. Hazard ratios for incident atrial fibrillation by levels of mtDNA copy number in pre-specified subgroups. Age groups were ≤65 and >65 for ARIC and MESA, ≤70 and >70 for CHS. Hazard ratios are for comparing the 10th to the 90th percentile of mtDNA copy number. Models were adjusted for age, sex, race/enrollment center, body mass index, height, smoking, alcohol intake, physical activity, total and HDL cholesterol, cholesterol medication, hypertension, diabetes, prevalent CHD, prevalent heart failure, eGFR and log-transformed NT-proBNP at baseline. Table S1. Baseline characteristics of study participants by mtDNA-CN quintiles in ARIC. Table S2. Baseline characteristics of study participants by mtDNA-CN quintiles in MESA. Table S3. Baseline characteristics of study participants by mtDNA-CN quintiles in CHS. Table S4. Hazard ratios for incident AF by quintiles of mtDNA copy number, among Black and White MESA participants. Table S5. Hazard ratios for incident AF by quintiles of mtDNA copy number in ARIC, using multiple imputation by chained equations (MICE) to impute for missing mtDNA-CN. Table S6. Hazard ratios for incident AF by quintiles of mtDNA copy number, using inverse probability weighting to account for death as dependent censoring. [file 12916_2020_1715_MOESM1_ESM.docx]
